# Immunogenomic Biomarkers and Validation in Lynch Syndrome

**DOI:** 10.3390/cells12030491

**Published:** 2023-02-02

**Authors:** Ramadhani Chambuso, Mbali Mthembu, Eveline Kaambo, Barbara Robertson, Raj Ramesar

**Affiliations:** 1 MRC Unit for Genomic and Precision Medicine, Division of Human Genetics, Department of Pathology, Institute for Infectious Diseases and Molecular Medicine, Faculty of Health Sciences, University of Cape Town, Observatory, Cape Town 7704, South Africa; 2 Department of Biochemistry and Medical Microbiology, School of Medicine, University of Namibia, 340, Windhoek 9000, Namibia; 3 Division of Medical Virology, Department of Pathology, Institute for Infectious Diseases and Molecular Medicine, Faculty of Health Sciences, University of Cape Town, Cape Town 7704, South Africa; 4 Department of Radiation Oncology, Groote Schuur Hospital, University of Cape Town, Observatory, Cape Town 7704, South Africa

**Keywords:** Lynch syndrome variant heterozygotes, colorectal and non-colorectal cancers, frameshift mutations, neoantigens, immune responses, immunogenomic biomarkers

## Abstract

Lynch syndrome (LS) is an inherited disorder in which affected individuals have a significantly higher-than-average risk of developing colorectal and non-colorectal cancers, often before the age of 50 years. In LS, mutations in DNA repair genes lead to a dysfunctional post-replication repair system. As a result, the unrepaired errors in coding regions of the genome produce novel proteins, called neoantigens. Neoantigens are recognised by the immune system as foreign and trigger an immune response. Due to the invasive nature of cancer screening tests, universal cancer screening guidelines unique for LS (primarily colonoscopy) are poorly adhered to by LS variant heterozygotes (LSVH). Currently, it is unclear whether immunogenomic components produced as a result of neoantigen formation can be used as novel biomarkers in LS. We hypothesise that: (i) LSVH produce measurable and dynamic immunogenomic components in blood, and (ii) these quantifiable immunogenomic components correlate with cancer onset and stage. Here, we discuss the feasibility to: (a) identify personalised novel immunogenomic biomarkers and (b) validate these biomarkers in various clinical scenarios in LSVH.

## 1. Introduction

Lynch syndrome (LS) is the most common inherited cancer predisposition syndrome caused by germline pathogenic variants (PV) in the DNA-mismatch repair (*path*_MMR) genes, *MLH1*, *MSH2*, *MSH6*, and *PMS2*, or by deletions in *EPCAM* [1,2]. Approximately 10–15% of early-age-onset colorectal cancer (CRC) is attributable to LS, which has a prevalence of 1 in 280 people in Australia, Canada, and USA populations [3]. CRC, which is a traditional hallmark cancer of LS, accounts for up to 80% of primary tumour sites [4,5]. CRC is commonly prevented and cured by screening, surveillance (mainly by colonoscopy), and modern surgical and medical treatments, with an average 10-year survival rate of 90% for stage I disease [6,7]. Approximately 5% to 10% of all CRC cases are caused by high penetrance familial cancer syndromes, including LS [8,9]. The majority of LS-variant heterozygotes (LSVH) develop colorectal cancer, amongst other cancers, including endometrial, ovarian and prostate cancers [10]. The incidence of multiple cancers in other body organs is also higher in LSVH than in the general population [11]. The higher mortality rate is of concern in developing countries notably due to poor surveillance and a lack of early-stage screening methods, late diagnosis, and inadequate/inappropriate treatment [12].

Identification of individuals with hereditary cancers, including their surveillance based on empiric risks, leads to improved cancer outcomes [13,14]. More specifically, ascertainment of families with LS (in which there are no premonitory lesions) and the identification of PV and best-practice clinical surveillance dramatically reduces morbidity and mortality [13,14,15]. The recommended frequency, invasiveness, and procedure-associated risks of colonoscopies (and other cancer-screening tests) are known to influence adherence to and compliance with cancer-screening guidelines [16,17,18,19]. Existing screening and surveillance guidelines for LSVH are based on average age-specific cumulative cancer risk [20].

Furthermore, these guidelines fail to consider the fact that there is substantial heterogeneity between LSVH with different PV, which presents challenges for diagnosis and management [21,22]. Detection of tumour-derived, cell-free, nucleic acids in stool and blood has emerged as a promising biomarker in gastrointestinal cancers, and various assays for their detection have been developed. These include the stool-based DNA multi-marker (ColoGuard^®^) or the blood-based assay for methylated Septin 9 DNA [23,24]. However, even with extremely sensitive techniques, most early-stage tumours and precancerous lesions do not release detectable amounts of circulating tumour DNA especially for non-colorectal cancers [25,26,27]. Due to a lack of prospective data, the current guidelines also rely on invasive cancer screening tests and retrospective data from patient cohorts whose molecular testing was initially biased and generalised using a “one-size-fits-all” approach for every LSVH [8,28]. Therefore, there is a need to identify alternative, personalised, non-invasive means for detecting and monitoring for the development of both premalignant and malignant lesions with high sensitivity and specificity in LSVH [16,17,29].

Through genetic mutations or epigenetic silencing, MMR-deficiency (dMMR) significantly increases the genomic mutation rate and predisposes LSVH to a remarkably higher-than-average risk and an excess incidence for all types of cancers [30]. Despite an extremely good recovery rate in first cancers, LSVH often develop more lethal cancers at a relatively young age [31]. This highlights the need for better molecular evaluation and identification of patients who require more intensive molecular and clinical surveillance. Personalised medicine plays an important role in managing patients, particularly patients with PV in genes that predispose them to cancer [32].

LS cancers have a high mutational burden that results in a defined set of frameshift peptide neoantigens [33]. Based on the increasing knowledge of the mutational landscapes of cancers with dMMR, it can be predicted that mutant neoantigens trigger strong immune responses by CD8+ cytotoxic T cells functioning as major mediators of anti-cancer immunity [29,34]. Insertion and deletion mutations in microsatellites occur during DNA replication, and the failure to repair the mutations due to the dMMR phenotype contributes to tumorigenesis [35]. The induced shift in the protein reading frame generates neoantigens that are recognised as foreign by the immune system [34]. T-cell immune responses specific to the frameshift peptides (FSPs) have previously been observed in the peripheral blood of both LSVH with CRC and in LSVH that had never developed cancer or adenomas [36,37,38]. Immune responses such as these suggest that the immune system has been pre-symptomatically exposed to FSPs generated by dMMR cells during life [29,39]. Furthermore, there is a high prevalence of non-neoplastic or early dysplastic dMMR cells in the intestine and other organs of LSVH, as well as mutations causing FSPs in the colonic crypts [33]. In addition, LS has been associated with marked local immune responses, including LS-related CRCs, adenomas, dMMR crypts, and even completely normal-appearing colonic mucosa [29,33,40]. All of these observations suggest that the adaptive immune system plays a critical role in suppressing and controlling the growth of dMMR cancers in the host [29,41].

We hypothesise that the presence of PV and expression of neoantigens in LS generate measurable and dynamic immune components that may correlate with cancer initiation and/or progression [29,42]. If effectively characterised, this phenomenon could be used as an alternative to the regular invasive cancer screening tests and as novel immunogenomic biomarkers for early cancer detection, progression or control in LSVH.

## 2. Frameshift Neopeptides, Neoantigens, and the Immune Responses in LS

**Figure 1:**
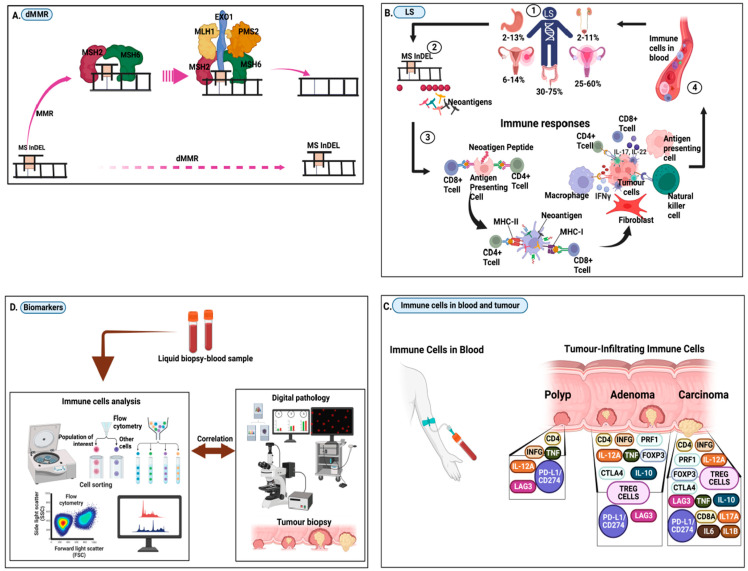
Schematic illustration of pathogenesis and characterisation of immunogenomic biomarkers in blood. (**A**) Deficiency in mismatch repair (dMMR) occurs when the microsatellite (MS) indel is not repaired by MMR genes. (**B**) LS, which occurs due to dMMR. When MS indels are not repaired in LSVH, frameshift neopeptides are formed and accumulate on the cell surface and in the bloodstream as neoantigens. These neoantigens trigger immune responses that accumulate in the circulation and infiltrate the neoplasm during early carcinogenesis. (**C**) Immune cells (including pro-inflammatory and checkpoint molecules) in blood and tumour-infiltrating cells correlating with different neoplasm/tumour stages (**D**) Immunogenomic biomarker analysis to detect cellular and acellular immune components in blood. Biomarker profiles correlated with clinical parameters in digital pathology results for cancer diagnosis and prognosis assessments.

When cancers with dMMR develop, they accumulate a large number of mutations [43,44]. Physiologically, the MMR system detects and corrects base mismatches caused by polymerase slippage during DNA replication. Microsatellites are repeated sequence segments that are frequently affected by these mismatches. Uncorrected mismatches lead to the accumulation of insertion/deletion mutations (indels) in dMMR cells [33,44,45] (Figure 1A). Indels of specific coding microsatellites (MS) located within tumour suppressor genes, particularly *TGFBR2* and *ACVR2*, are major causes of malignant transformation and cancer progression of dMMR cells [33,44]. MS indels are functionally significant, and their distribution in manifested dMMR cancers is not random but follows Darwinian principles of selection [33]. Recurrent MS indels however are well known and have been documented in many independent studies for different types of dMMR tumours [45]. Mutations such as these not only inactivate tumour-suppressive signalling pathways but also cause a shift in the translational reading frame, resulting in novel FSPs as neoantigens [45]. The accumulation of frameshift mutations in genes comprising coding microsatellites (cMS) is favoured by dMMR. Due to mutation-induced frameshift peptides (neoantigens), microsatellite unstable (MSI) cancers are highly immunogenic. Nearly all MSI cancers express the same set of neoantigens, which are the result of functionally relevant driver mutations [46] (Figure 1B). As opposed to point mutations, which lead to the alteration of single amino acids, indel-mediated frameshifts give rise to long segments of amino acid sequences that are completely foreign to the host immune system [33,44,45]. As a result, the immunogenicity of dMMR cancers is not only due to the sheer number of somatic mutations but also to the number of potential epitopes in FSPs caused by indel mutations [44].

In addition to an already marked immune activation in LSVH (as a result of new coding mutations arising in every cycle of cell division), dMMR further generates frameshift mutations that lead to highly immunogenic neoantigens that trigger an immune response in the body [29,34,36]. Specific immune responses against these neoantigens are triggered in LSVH. Neoantigens are proteins specific to tumours that are not expressed in normal cells. Because these neoantigens are selectively expressed on tumours, they may minimize immune tolerance and the risk of autoimmunity [47]. Recent evidence shows that neoantigens are recognized by the immune system and can be targeted to enhance anti-tumour immunity. In addition, tumours with low mutational loads continue to express neoantigens and are susceptible to some forms of immune attack [46,47]. A cancer antigen is an antigen recognized by the immune system and produced when the cancer genome is altered, including cancer testis antigen and neoantigens. The formation of neoantigens can be caused by mutations in cancer cells that alter the proteins from ‘self’ [48,49].

The association between strong immunogenicity and dMMR is generally explained by the accumulation of frameshift mutations within runs of coding mononucleotide sequences and the synthesis of neoantigens [36,50,51]. Neoantigens can trigger the immune system to launch an attack against the cells producing these proteins (Figure 1B) [34,36,52]. Neoantigens are also capable of eliciting a CD4^+^ T cell-specific response in addition to CD8^+^ T cells, while T lymphocytes play a critical role in controlling cancer progression (Figure 1B) [29,34,53,54]. In our recent work [55], we observed that cancers attributable to HIV/HPV infection were the least reported histologically confirmed cancers in a large European cohort of LSVH [56]. Previously, we hypothesised that LSVH may control a range of acute and chronic infections, including HIV/HPV-infections, and perhaps also cancers attributable to these infections. We originally theorised that this may be due to the continuous hyperinflammatory status of the immune system in LSVH caused by the uninterrupted production of neoantigens [29,33,40].

Increased density of tumour-infiltrating lymphocytes and heightened T-cell responses are a cardinal feature of LS [34]. Moreover, mutated cancer proteins are known to elicit strong antitumour-T-cell responses that correlate with clinical findings [36,57]. Neoantigen degradation releases immunogenic neo-peptides on the surface of tumour cells presented by human leukocyte antigen class I (HLA-I) molecules, against which a specific CD8+ T-cell immune response is directed (Figure 1B) [34,51]. There are a variety of genomic mutations that can lead to the formation of neoantigens, including non-synonymous mutations, retained introns, post-translational modifications that alter amino acids, gene fusions, and frameshift-in/del variants [49]. A major histocompatibility complex protein (MHC) may bind these novel peptides during normal protein degradation and present them on the cell surface as neoantigens (i.e., tumour-specific peptides that are recognized by the immune system as non-self and cause cancer cell destruction) [58,59]. There are inherited, sporadic, and somatic genetic contributions to genome instability that often interact to promote cancer through a variety of effects on genome instability that can be detected by various diagnostic approaches [60]. Identification of these alterations not only provides clues to newer cancer therapies that can use the patient’s immune system to eradicate the disease, it is also important in the context of cancer predisposition, surveillance and early detection [48]. Immune checkpoint blockade inhibitors, known as therapeutic cancer treatments, are associated with genomic instability and neoantigen formation [60].

In addition, both cell-mediated and humoral responses of the immune system are also central to inflammation influencing tumorigenesis [61,62]. The observation of an elevated immune system in LSVH, as a response to neoantigens generated by cells acquiring secondary (unrepaired) mutations during the replication process and inflammation during carcinogenesis, warrants attention as a potential means for monitoring cancer development in pre-symptomatic LSVH at the molecular level (using immunogenomic biomarkers) [36,44,63,64].

In a previous study [36], which included both healthy LS patients without a history of cancer and LSVH with CRC, FSP-specific effector T cells were detected in peripheral blood. Analysis of the immune responses in these individuals revealed that the observed T-cell responses were directed toward 14 different FSP antigens predicted from human genome databases. It has been previously suggested that tumour-specific FSPs are responsible for the high immunogenicity of dMMR tumours. In an ELISpot analysis to determine reactivity against 14 predicted FSPs in MSI-H CRC patients, CD3/CD28-expanded TiTc from these patients reacted strongly against several of the selected MSI-induced FSPs. These antigens exhibited different mutation frequencies. In another study, a number of neoantigens derived from genes with high mutation frequencies that exhibited immunogenic properties in vitro were also found in LS [60].

The activation of immune responses against neopeptides in healthy LS mutation carriers without a history of tumour development can be explained by the generation of frameshift peptides already in haploinsufficiency when a dMMR gene becomes relevant. In LSVH, the type and intensity of the infiltrating immune cells may reflect the pathological tumour stage [65]. It is also possible that CD8^+^ T-cell immune responses predict outcome in early-stage tumours, as the immunogenomic load correlates with cancer outcomes in LSVH (Figure 1C) [65]. This implies that immune responses may not only be a predictor but also a potential means to intervene in cancer development as immunogenomic biomarker profiles correlate with clinical parameters and pathology results for cancer diagnosis and prognosis assessments (Figure 1C) [66].

## 3. Research Questions in Lynch Syndrome Immunogenomic Biomarkers

(i) How can the measurable immunogenomic components in LSVH that occur as a result of mutations in the mismatch-repair genes be characterised?

Rationale:

LS cancers are characterised by a higher-than-average burden of mutational frameshift neoantigens, which trigger a large pool of immune response components in the bloodstream [29,33]. To date, there have been no previous approaches that followed this logic, either by studying recurrent FSPs derived from functionally relevant driver mutations or by evaluating dynamic immune components responsive to frameshift neoantigens in blood as immunogenomic biomarkers for high-sensitivity cancer detection in LS [29,66].

(ii) Could immunogenomic biomarker profiles serve endophenotypically as potential biomarkers to reflect neoplastic changes (from early-stage to invasive and metastatic cancer) in LSVH?

**Figure 2:**
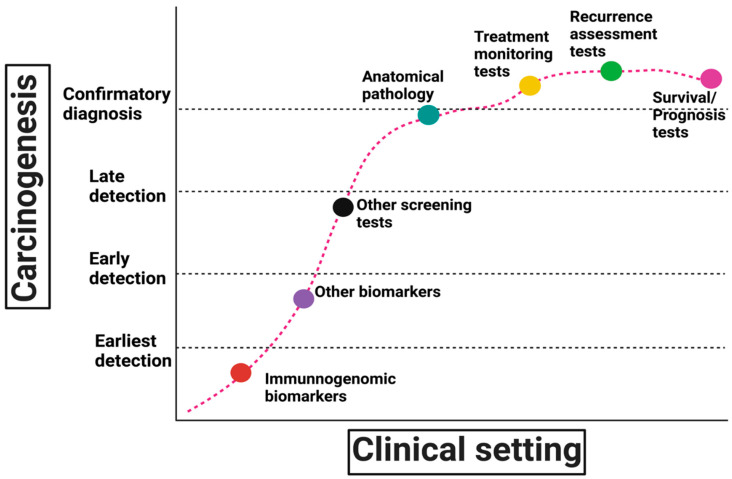
Clinical applications of immunogenomic biomarkers in carcinogenesis. In LS, immunogenomic biomarkers could be more sensitive for detecting malignancies than conventional imaging or other approaches. This sensitivity can be exploited in several ways, such as detecting cancers in LSVH before symptoms or radiological manifestations appear and detecting minimal residual disease. As an alternative to invasive surveillance and cancer screening, immunogenomic biomarkers can be used to screen for cancer even in the absence of other clinical evidence. They can also be used to assess cancer prognosis in patients with LS who have completed all potentially curative therapies. In patients with radiographically detectable disease, immunogenomic biomarkers may also be more sensitive for tailored monitoring of tumour response (treatment monitoring tests).

Rationale:

In a previous study, healthy LSVH and LS patients with CRC were shown to have an FSP-specific effector T cell population in peripheral blood [36]. Several neoantigens from genes with high mutation frequency that showed immunogenic properties in vitro were also found in healthy LSVH. The immune responses may suggest a pathological tumour stage in LSVH [65]. In addition, the burden of blood immune responses may correlate with cancer initiation in the earliest stages for clinical applications of immunogenomic biomarkers [29] (Figure 2). Since immunogenomic biomarker profiles may correlate with clinical parameters and pathology outcomes for cancer diagnosis and prognosis, this implies that immune responses may serve not only as a predictor but also as a means to intervene in cancer development [29,66] (Table 1).

(iii) Can immunogenomic biomarker profiles serve to prognosticate, i.e., predict disease-free survival and overall survival for LSVH carrying the same or different novel PV?

Rationale:

Generally, certain aspects of the immune profile can predict a patient’s cancer prognosis [29,66]. However, it has proven difficult to establish a standard prognostic criterion for LSVH. Alternatively, analysis of immunogenomic biomarkers in prospective cohort studies may be able to answer this question. It is possible to predict cancer prognosis, survival and treatment response in LSVH using immunogenomic biomarkers alone or in combination with other factors/biomarkers (Table 1). In addition, immunogenomic biomarker profiling can be used to build a prognostic model to provide clinicians with simple tools to accurately predict prognosis and treatment outcomes of cancer in LSVH [29,67], or simple evaluation of systemic immune-inflammation index as biomarkers in LSVH, an example of which is from urinary system cancers [68]. In addition, longitudinal studies can be used where blood samples are available at presymptomatic genetic diagnosis and at various (surveillance) follow-up times. Comparison of biomarkers for risk assessment is necessary because high levels of immunogenomic biomarkers may predict the risk of recurrence or poor prognosis of the disease [69].

**Table 1:** Different panels of immunogenomic biomarkers and other diagnostic/prognostic biomarkers in cancer.

1. IMMUNOGENOMIC BIOMARKERS	2. OTHER BIOMARKERS
**Panel 1 biomarker profiles (for polyp)** CD4,INFG, LAG3,PDL1,/CD274, IL12A, TNF [34]	**A. Specific for colorectal cancer** (i) Faecal occult blood testing (ii) Stool DNA, miRNA [24,70] (iii) Faecal immunological test (FIT) [71] (iv) Faecal bacteria (v) Gut microbiota signatures [72]
**Panel 2 biomarker profiles (for adenoma)** PRF1,FOXP3,CTLA4, IL-10,TREG CELLS [34]	**B. Colorectal and non-colorectal cancers** (i) DNA, RNA, cfDNA, ctDNA, cfRNA, mRNA, microRNA, IncRNA (ii) Circulating tumour cells (iii) CA 125 Blood test [73] (iv) Methylation tests [23] (v) Growth factors tests (vi) Tissue tests (vii) Proteins and Glycoproteins tests [74] (viii) Tissue tests (ix) Volatile organic compounds (VOC) [75] (x) Immune-Inflammation index [68] (xi) Prostate cancer antigen 3 test (PCA3) [76] (xii) Genomic Prostate Score [77]
**Panel 3 biomarker profiles (for carcinoma)** CD8A, IL17A, IL1B, IL6 [34]	

## 4. Validation of Immunogenomic Biomarkers

Immunogenomic biomarker panels can be compared with colonoscopy tests, which are the current standard of care for CRC clinical surveillance. The LSVH patient will be asked to provide blood before a colonoscopy or even before preparation for a colonoscopy. As a single marker and in combination, the sensitivity and specificity of immunogenomic biomarkers to identify high-grade dysplasia or adenoma during screening will be estimated. Subjects with no symptoms who undergo a colonoscopy to detect CRC are eligible for the procedure. After colonoscopy, the clinical findings will be correlated with the levels of immunogenomic biomarker panels. For extracolonic cancers, the levels of immunogenomic biomarkers will be correlated with histopathology findings [78,79].

For the detection of invasive colorectal neoplasms and for the screening of relevant neoplasms, we will estimate the sensitivity and specificity of immunogenomic biomarkers in the blood and their confidence intervals. We will then test the primary hypothesis to confirm whether a particular biomarker test or panel is clinically accurate. Data analysis will be guided by state-of-the-art information about candidate biomarkers and pathology tests available at the time of the analysis. A variety of primary hypotheses are provided in order to justify the size of the study sample. The final step will be the evaluation of several alternative tests and multi-marker panels. As a secondary analysis, we will examine how individual heterogeneity affects marker performance. In order to identify immunogenomic biomarkers for early detection of cancer in blood samples collected from LS patients diagnosed with cancer, those with an LSVH diagnosis, and healthy individuals, a biobank collection of appropriately preserved blood and biopsy biospecimens will be created for future validation and additional biomarker discovery [79,80].

Biomarker analytical validation is the process of determining how accurately and reliably a test measures what analytes are of interest in a patient blood sample. Pre-analytical, analytical, and post-analytical phases comprise the analytical validity of an assay. Validation should demonstrate how robustly and reliably the test results correlate with the clinical outcome of interest. In practice, clinical validity implies that the cancer biomarker assay can distinguish two or more distinct groups with different biological characteristics. An assay’s clinical utility is its ability to improve clinical outcomes and show if the biomarker/s improve patient outcomes or add value to healthcare decision-making compared with current practice [81,82].

### 4.1. Pre-Analytical Validation

The evaluation of pre-analytical factors that may affect assay performance due to specimen-related variability is an important step in biomarker validation. A standard operating procedure (SOP) to control specific biomarker development steps is essential to ensure optimal preanalytical processing. Optimization protocols for blood collection and storage media are often developed in conjunction with other pre-analytical parameters to establish best practice metrics. Guidelines for pre-analytical quality indicators and harmonization of analytical stability and laboratory quality control (QC) were published in a similar previous study [83].

It is recommended that the following validation practices/steps be followed:

i. Pre-assessment of biomarkers and ensuring an expedient approach to assay development;
ii. Consider quality assurance and quality control procedures for blood-based assays for each specific biomarker panel;
iii. Maintain optimal pre-analytical processing procedures and SOPs for control of specific biomarkers;
iv. During analytical validation, it is advisable to use procedures that include strict quality assurance, reproducibility protocols, and control procedures;
v. Whenever possible, reagents and assay controls (positive and negative controls) must be included in the interpretation of assay results;
vi. Biostatistical and computational approaches to quantification and interpretation of data must be considered. In addition, the development of algorithms for multiplex signatures based on phenotypic, functional, and genomic data must be considered;
vii. An integrated bioinformatics approach needs to be considered for the integration of complex high-throughput immunogenomic data consisting of multiple components;
viii. The use of reference standards and/or coordination efforts between centralized laboratories (proficiency panels) is recommended to assess the robustness of semi-quantitative methods and to enable analytical and clinical validation of biomarkers.

### 4.2. Outcome Measures

A. Primary outcome measure
  To test the sensitivity of a blood-based panel of immunogenomic biomarkers against colonoscopy and other cancer screening methods. A consensus panel based on blood samples will be able to detect colorectal adenocarcinoma with a significant improvement in sensitivity and specificity [80,84]. However, the persistence of immunogenomic biomarkers in the blood of already diagnosed and treated patients may affect the specificity of the biomarkers. This is because they may remain at high levels even after treatment for cancer or precancerous lesions.
B. Secondary Outcome Measures
  i. To test the specificity of blood-based immunogenomic biomarkers compared with pathology tests [80], using blood-based panels to detect colorectal adenocarcinoma with the same or higher sensitivity as pathology tests. The hypothesis is that they have a significantly higher specificity than 0.55 with an expected specificity greater than 0.70.
  ii. To analyse the sensitivity and specificity of a combined panel of blood-based immunogenomic biomarkers for CRC detection [80,84]. To test the hypothesis that combining the blood-based panel with the tissue-based panel will improve the detection of colorectal adenocarcinoma: with a sensitivity greater than 0.98, it will have a specificity of 0.55 or greater.
  Using the area under the receiver operating characteristic (ROC) curves and logistic regression models, models that have high sensitivity, specificity, positive predictive value, and negative predictive value for advanced neoplasms detection compared to healthy individuals were found [85]. By multiplex flow cytometry, which examines a range of lymphocyte markers, phenotypic analysis of T cells can provide information about their activation status. A baseline signature of immune cells and pro-inflammatory markers with a higher baseline frequency of CD4^+^CD25^+^FoxP3^+^ Tregs should be identified. For this immunogenomic biomarker signature to be used in routine clinical practice, it needs to be analytically and clinically validated (including a panel of markers required to analyse and enumerate cells).

## 5. Conclusions

It is possible to identify measurable and dynamic immunogenomic components that, if effectively characterised, can be used as novel diagnostic and prognostic biomarkers to monitor cancer progression in both healthy and cancer diagnosed LSVH as an alternative to invasive cancer screening tests, including colonoscopies. We have highlighted all major steps of the entire biomarker validation process, including: (i) analytical validation, (ii) clinical validation, (iii) clinical utility demonstration strategies, and (iv) applicable diagnostics related to immune response cell assays. The use of immunogenomics in LSVH may provide (i) novel and personalised immunogenomic biomarkers for non-invasive cancer screening and surveillance, (ii) fewer invasive colonoscopies for CRC screening and diagnosis, and (iii) novel insights into cancer immunotherapies and vaccine development. Ongoing and future vaccine immunoprevention studies in LS will guide the development of technologies that can then be applied more broadly to help these individuals with a higher-than-average risk of cancer.

## Data Availability

Not available.
